# The First Reported Albanian Patient With Fructose-1,6-Bisphosphatase Deficiency: A Rare Disorder of Fructose Metabolism

**DOI:** 10.1155/carm/4567735

**Published:** 2025-09-09

**Authors:** Paskal Cullufi, Gladiola Hoxha, Inva Gjeta, Ermira Dervishi, Virtut Velmishi, Eda Jazexhiu-Postoli, Ermira Kola, Sonila Tomori, Durim Sala, Mirela Tabaku

**Affiliations:** ^1^Pediatric Department, University Hospital Center “Mother Teresa”, Tirana, Albania; ^2^Neonatology Department, “Hygeia” Hospital, University of Medicine Tirana, Tirana, Albania; ^3^Pediatric Department, Genetic Service, University of Medicine, Tirana, Albania

## Abstract

**Background:** Fructose-1,6-bisphosphatase (FBPase) deficiency is a rare autosomal recessive disorder of gluconeogenesis caused by biallelic pathogenic variants in the *FBP1* gene. It presents with episodic hypoglycemia, lactic acidosis, and ketone body abnormalities, particularly during catabolic stress, but often mimics more common metabolic disorders, leading to diagnostic delays.

**Case Presentation:** We describe the first genetically confirmed Albanian case of FBPase deficiency in a 3-year-old girl, born to nonsanguineous parents. The patient presented with recurrent episodes of vomiting, hypoglycemia, and metabolic decompensation since infancy. At her most severe presentation, she was admitted in a subcomatose state with profound hypoglycemia (35 mg/dL) and lactic acidosis (pH 6.9) without ketonuria. Whole exome sequencing identified a homozygous pathogenic *FBP1* variant NM_000507.3(FBP1): c.472C > T; p. (Arg158Trp), a recurrent missense mutation associated with significant phenotypic variability. Parental testing confirmed autosomal recessive inheritance.

**Management and Outcome:** Emergency management included intravenous dextrose and bicarbonate for metabolic acidosis, followed by nutritional interventions. The patient was advised to avoid fasting for more than 8 h and to limit fructose intake. No further metabolic crises were observed after these interventions.

**Conclusion:** This case highlights the clinical and genetic complexity of FBPase deficiency and underlines the importance of genomic diagnostics in children with unexplained hypoglycemia and metabolic acidosis. Early diagnosis allows effective dietary management and prevents recurrent life-threatening episodes. As the first reported case in Albania, it contributes to the growing recognition of FBPase deficiency as an underdiagnosed but treatable metabolic disorder.

## 1. Introduction

Fructose-1,6-bisphosphatase (FBPase) deficiency is an autosomal recessive metabolic disorder caused by mutations in the *FBP1* gene [1]. Baker and Winegrad first described the clinical features of the disease in 1970 [2], and in 1995, Kikawa et al. identified three pathogenic mutations in the *FBP1* gene associated with the disease [3]. The *FBP1* gene is located on Chromosome 9q22.2-q22.3 [4].

To date, more than 100 pathogenic or likely pathogenic variants in the *FBP1* gene have been reported [5]. FBPase deficiency is an extremely rare inherited disorder with an estimated incidence of approximately 1 in 350,000 to 1 in 900,000 live births [6]. Due to its low incidence, understanding of the underlying molecular mechanisms and genotype–phenotype relationships remains limited. In particular, adult presentations are rare and less well understood [7].

FBPase is a key regulatory enzyme in gluconeogenesis, catalyzing the hydrolysis of fructose 1,6-bisphosphate to fructose 6-phosphate. This step is critical for endogenous glucose production from gluconeogenic substrates such as alanine, glycine, glycerol, and lactate/pyruvate [4]. Consequently, affected patients often present with hypoglycemia when glycogen stores are depleted, particularly during fasting, infection, or catabolic stress—especially in the neonatal period [4].

Nearly half of the affected infants experience hypoglycemia in the first 4 days of life due to inadequate glycogen stores [5]. In adults, where glycogen stores are more developed, the impact of these triggers tends to be less pronounced [8]. Clinical manifestations usually include intermittent metabolic crises presenting as lactic acidosis and ketotic hypoglycemia, which may progress to life-threatening symptoms such as hyperventilation, apnea, seizures, or coma.

In this report, we describe the clinical history of a 3-year-old girl with FBPase deficiency who, prior to diagnosis, had recurrent episodes of vomiting and hypoglycemia without ketosis.

## 2. Case Report

A 3-year-old girl was admitted to the pediatric intensive care unit (PICU) with severe dehydration and a subcomatose state. Her parents reported several episodes of vomiting, abdominal pain, generalized weakness, loss of appetite, drowsiness, and respiratory distress. She had also been febrile for the previous 2 days, with a maximum-recorded temperature of 38.5°C.

The patient was born at term after an uneventful pregnancy. Apgar scores at 1 and 5 min were 9 and 10, respectively, and her birth weight was 3500 g. She had experienced three separate episodes of vomiting and hypoglycemia. During the first episode, she was monitored for 6 h and given intravenous fluids. For the second episode, she was admitted to hospital with viral gastroenteritis and treated with intravenous fluids for 72 h.

### 2.1. Physical Examination

Physical examination on admission revealed a critically ill child. Neurological assessment using the Glasgow Coma Scale (GCS) was 10. Pupils were equal, isochoric, and reactive to light. The skin was pale, with reduced turgor and no rash or pathological findings. There were signs of circulatory shock: tachycardia (180 bpm), weak radial pulse, and a prolonged capillary refill time of 4-5 s. Blood pressure was markedly reduced (60/30 mmHg). The patient had rapid, deep, acidotic respiration (respiratory rate: 40 breaths per minute). Lung auscultation was normal without adventitious sounds. Abdominal examination revealed a soft, nontender abdomen with a palpable liver 3-4 cm below the costal margin. The spleen was not palpable, and there was no lymphadenopathy.

### 2.2. Laboratory Evaluations

The data at the time of admission and after 48 h are presented in Table 1.

Initial investigations included blood gases, lactic acid, plasma glucose, liver function tests, a coagulation profile, and urinary ketones. Urinary ketones were repeatedly negative on dipstick analysis. Urine organic acid analysis was not performed due to limited access during the acute episode. The patient exhibited high anion gap metabolic acidosis, with an anion gap of 30.9 mEq/L (Anion Gap = ([Na^+^] + [K^+^]) − ([Cl^−^] + [HCO_3_^−^]) = (141 + 4) − (110 + 4.1) = 30.9 mEq/L). As triglyceride levels were not measured, pseudo-hypertriglyceridemia could not be excluded. Extended metabolic testing, including plasma amino acids, acylcarnitine profiling, and gas chromatography–mass spectrometry (GC–MS), was unavailable at the time.

Based on the clinical and biochemical findings—severe hypoglycemia (defined by the WHO as plasma glucose < 40 mg/dL in symptomatic children), lactic acidosis, and mild hepatomegaly—the differential diagnosis included inborn errors of metabolism affecting either gluconeogenesis or mitochondrial energy metabolism. Specifically, glycogen storage diseases (GSDs), fatty acid oxidation disorders (FAODs), organic acidemias, and mitochondrial disorders were considered. Based on anamnestic data and the combination of symptoms, a defect in gluconeogenesis, particularly FBPase deficiency, was initially suggested.

Whole exome sequencing (WES) was performed on samples collected from the patient and her parents using dried blood spot (DBS) cards to confirm the diagnosis. WES and bioinformatic analysis were conducted at a certified reference laboratory in Germany, with variant interpretation following the guidelines of the American College of Medical Genetics and Genomics (ACMG). The identified variants were confirmed by Sanger sequencing. The results revealed a homozygous pathogenic variant in the *FBP1* gene. Genetic analysis revealed a homozygous missense variant in the *FBP1* gene: NM_000507.3 (FBP1): c.472C > T; p. (Arg158Trp), which is located in Exon 4. This nucleotide substitution results in the replacement of arginine with tryptophan at Codon 158, a highly conserved amino acid residue critical for enzymatic function. Targeted Sanger sequencing of both parents confirmed heterozygosity for the same variant, which is consistent with autosomal recessive inheritance. This confirms the patient's homozygosity for the pathogenic *FBP1* gene variant. These findings confirmed the molecular diagnosis of FBPase deficiency.

### 2.3. Acute Management and Follow-Up

Emergency treatment was started immediately. After a glucometer reading revealed severe hypoglycemia, the patient received a 10% glucose bolus (2 mL/kg over 5 min). This was followed by a normal saline bolus of 20 mL/kg. Due to persistent signs of poor perfusion, a further 20 mL/kg saline bolus was administered over 20 min. To maintain normal blood glucose levels, a 10% glucose infusion was started at a rate of 5 mL/kg/h, while calculating the fluid deficit and maintenance requirements. To correct the severe metabolic acidosis, sodium bicarbonate was administered according to the formula: (0.15 × body weight × base deficit in mmol/L). Initially, half the calculated dose was infused over a minimum of 30 min. Once the deficit and maintenance fluids were calculated, intravenous fluids consisting of 0.45% saline with 10% glucose were administered to correct the deficit over 24 h. Oral feeding was resumed once the patient's clinical status had improved and vomiting had resolved.

During the subsequent 12-month follow-up period, the child demonstrated steady weight gain, normal psychomotor development and no recurrence of metabolic crises. Dietary management included avoiding fasting for more than 8 h and moderating dietary fructose intake. These interventions resulted in stable metabolic control and an excellent clinical outcome.

## 3. Discussion

FBPase deficiency is a rare but potentially life-threatening autosomal recessive disorder of gluconeogenesis. It typically presents in infants and young children, although it can also present in neonates or at a later stage in life, depending on the severity. It is characterized by episodes of hypoglycemia, metabolic acidosis, and ketosis, which are often triggered by fasting or intercurrent infections [9].

Although the frequency of metabolic crises tends to decrease with age, early diagnosis remains crucial to prevent acute complications, long-term developmental delays, and hypoglycemia-related morbidity and mortality [10–12].

To date, approximately 150–200 cases have been documented worldwide [11]. Due to its nonspecific clinical presentation, FBPase deficiency is frequently misdiagnosed or diagnosed late. The clinical features may closely resemble other inborn errors of metabolism, such as GSDs, FAODs, and mitochondrial disorders [13].

In our patient, laboratory findings revealed severe metabolic acidosis accompanied by nonketotic hypoglycemia. Urinary ketones were repeatedly negative using dipstick analysis. However, it is acknowledged that urine keto-sticks may lack sensitivity, and blood or capillary ketone testing provides a more accurate assessment of ketone status. Unfortunately, blood ketone levels were not obtained during the acute episode. Nevertheless, we support the use of blood ketone measurements whenever possible, as they can significantly aid in the differential diagnosis of inborn errors of metabolism [14]. Although ketosis is commonly observed in FBPase deficiency, it may be absent in some cases. The absence of ketosis in some cases may be explained by the accumulation of pyruvate and increased levels of oxaloacetate, which direct acetyl-CoA into the tricarboxylic acid (TCA) cycle rather than ketogenesis. Moreover, elevated cytosolic malonyl-CoA may inhibit carnitine Palmitoyltransferase I, restricting mitochondrial fatty acid transport and consequently reducing ketone body production [9]. In clinical practice, the presence or absence of ketosis during hypoglycemia can provide valuable diagnostic clues. For instance, GSDs are typically associated with hepatomegaly and ketotic hypoglycemia, whereas FAODs more often present with nonketotic hypoglycemia [15]. GC–MS may be diagnostically valuable in such cases by detecting elevated levels of glycerol-3-phosphate—a biochemical marker suggestive of a gluconeogenic defect. This method should be considered for future evaluations when available [16]. A genotype–phenotype correlation study found that 73% of patients with FBP1 mutations exhibited ketosis [17]. Despite its rarity, FBPase deficiency is genetically heterogeneous. More than 100 pathogenic variants have been described in the coding region of the *FBP1* gene, including small insertions/deletions, missense, nonsense, and splice-site variants [5]. Notably, more than one-third is missense mutations [7, 18].

Our patient carried a homozygous missense mutation NM_000507.3 (FBP1): c.472C > T; p. (Arg158Trp) which substitutes arginine with tryptophan and disrupts enzymatic activity. This variant is among the most commonly reported worldwide and has been associated with considerable intrafamilial phenotypic variability, including differences in age of onset and severity of metabolic decompensation [18]. A recent genotype–phenotype study reported a mean age of onset of 14.97 months among patients homozygous for this variant [18], consistent with our patient's early presentation and delayed diagnosis. This highlights the importance of early genetic testing in cases of unexplained hypoglycemia and metabolic acidosis.

Early diagnosis of FBPase deficiency remains challenging and requires a high index of clinical suspicion supported by timely biochemical and molecular investigations. In most reported cases, diagnosis is delayed by months or even years. In our case, initial symptoms including vomiting and hypoglycemia emerged at 8 months of age, but the diagnosis was not confirmed until the age of three. Earlier application of genomic testing could have facilitated a timely diagnosis, allowing prompt dietary management and potentially avoiding repeated metabolic decompensation. Early diagnosis enables appropriate interventions that support normal growth and development and improve long-term outcomes [4].

Management of FBPase deficiency involves emergency correction of hypoglycemia and metabolic acidosis using intravenous glucose and bicarbonate, followed by preventive strategies. Long-term care includes avoidance of prolonged fasting, especially during febrile illnesses, through frequent feeding and the administration of slowly absorbed carbohydrates such as uncooked cornstarch. Unlike patients with hereditary fructose intolerance (HFI), individuals with FBPase deficiency can tolerate moderate amounts of fructose (up to 2 g/kg/day), provided intake is distributed evenly throughout the day [9].

With appropriate management, most patients demonstrate normal growth and development and improved fasting tolerance over time. The long-term prognosis is generally favorable when the condition is diagnosed early and managed effectively. It is important to educate caregivers to avoid fructose-containing syrups, particularly during illness, as many over-the-counter medications may contain hidden sources of fructose. This preventive measure may reduce the risk of hypoglycemic crises [19].

## 4. Conclusion

FBPase deficiency should be considered in any child presenting with hypoglycemic episodes precipitated by fasting, infection, or vomiting. Early genetic diagnosis is essential for establishing an accurate diagnosis and guiding appropriate treatment. With simple preventive measures—especially the avoidance of fasting—the prognosis is excellent, and psychomotor development is typically preserved. However, if left undiagnosed, affected individuals are at risk of prolonged and severe hypoglycemia, which may result in irreversible neurological damage or even death.

## Figures and Tables

**Table 1:** Laboratory results and other findings (on admission and after 48 h) with reference values.

Laboratory results	On admission	After 48 h	Ref values
RBC	4.57 × 10^6^/mm^3^	4.32 × 10^6^/mm^3^	4-5 × 10^6^/mm^3^
Hemoglobin	11.6 gr/dL	11 gr/dL	10.5–12.7 g/dL
WBC	15 × 10^3^/mm^3^	11 × 10^3^/mm^3^	4–12 × 10^3^/mm^3^
PLT	216 × 10^3^/mm^3^	225 × 10^3^/mm^3^	150–400 × 10^3^/mm^3^
Glucose	35 mg/dL	80 mg/dL	60–100 mg/dL
AST	51 U/L	78 U/L	21–44 U/L
ALT	41 U/L	65 U/L	9–30 U/L
Bilirubin level	0.76 mg/dL	0.58 mg/dL	0.3–1.0 mg/dL
BUN	32.2 mg/dL	28 mg/dL	7–20 mg/dL
Creatinine	0.76 mg/dL	0.8 mg/dL	0.44–0.84 mg/dL
PT	70%	75%	70–110
INR	1.23	1.22	0.85–1.15
aPTT	29 s	29.5	20–35 s
CRP	0.15	0.01	< 0.5 mg/dL
Ketonuria	Negative	Negative	< 0.6 mmol/L
PH	6.9	7.3	7.35–7.45
HCO_3_	4.1 mmol/L	15.3 mmol/L	20–25 mmol/L
PCO_2_	11.7 mmHg	32 mmHg	32–48 mm Hg
BE	−26 mmol/L	−8.3 mmol/L	−4.4 mmol/L
Lactic acid	9.5 mmol/L	3.1 mmol/L	0.2–1.8 mmol/L
Natrium	141 mmol/L	139 mmol/L	139–145
Potassium	4 mmol/L	4.4 mmol/L	3.5–5
Chloride	110 mmol/L	105 mmol/L	98–107
Cortisol level	Normal		2.19–12.66 μg/dL
Other findings			
Abdominal US	Mild hepatomegaly		
Cardiac US & ECG	Normal exams		
Chest X-Ray	Normal		

## References

[1] Chandrasekhar V., Yelkur P., K V. (2024). Infantile Fructose-1,6-Bisphosphatase Deficiency Masquerading as Mitochondriopathy. *Cureus*.

[2] Baker L., Winegrad A. I. (1970). Fasting Hypoglycaemia and Metabolic Acidosis Associated With Deficiency of Hepatic Fructose-1,6-Diphosphatase Activity. *Lancet*.

[3] Kikawa Y., Inuzuka M., Jin B. Y. (1995). Identification of Genetic Mutations in Japanese Patients With Fructose-1,6-bisphosphatase Deficiency. *The American Journal of Human Genetics*.

[4] Moey L. H., Abdul Azize N. A., Yakob Y. (2018). Fructose-1,6-Bisphosphatase Deficiency as a Cause of Recurrent Hypoglycemia and Metabolic Acidosis: Clinical and Molecular Findings in Malaysian Patients. *Pediatrics & Neonatology*.

[5] Fawdry H., Gorrigan R., Ramachandran R., Drake W. M. (2022). A Novel Variant of fructose-1,6-Bisphosphatase Gene Identified in an Adult With Newly Diagnosed Hepatitis C. *JIMD Reports*.

[6] Lebigot E., Brassier A., Zater M. (2015). Fructose 1,6-bisphosphatase Deficiency: Clinical, Biochemical and Genetic Features in French Patients. *Journal of Inherited Metabolic Disease*.

[7] Sakuma I., Nagano H., Hashimoto N. (2023). Identification of Genotype-Biochemical Phenotype Correlations Associated With Fructose 1,6-Bisphosphatase Deficiency. *Communications Biology*.

[8] Bijarnia-Mahay S., Bhatia S., Arora V., Adam M. P., Feldman J., Mirzaa G. M., Amemiya A., Pagon R. A. (2025). Fructose-1,6-Bisphosphatase Deficiency. *Genereviews® [Internet]*.

[9] Steinmann B., Santer R., van den Berghe G., Fernandes J., Saudubray J. M., van den Berghe G., Walter J. H. (2006). Disorders of Fructose Metabolism. *Inborn Metabolic Diseases*.

[10] Nyhan W. L., Al-Aqeel A. (2012). *Atlas of Inherited Metabolic Diseases*.

[11] Bhai P., Bijarnia-Mahay S., Puri R. D. (2018). Clinical and Molecular Characterization of Indian Patients With Fructose-1,6-Bisphosphatase Deficiency: Identification of a Frequent Variant (E281K). *Annals of Human Genetics*.

[12] Ergoren M. C., Tuncel G., Sag S. O., Temel S. G. (2020). A Rare Case of Fructose-1,6-Bisphosphatase Deficiency: A Delayed Diagnosis Story. *Turkish Journal of Biochemistry*.

[13] Gaignard P., Eyer D., Lebigot E. (2017). UQCRC2 Mutation in a Patient With Mitochondrial Complex III Deficiency Causing Recurrent Liver Failure, Lactic Acidosis and Hypoglycemia. *Journal of Human Genetics*.

[14] Fujimoto S., Kobayashi M. (2020). Clinical Utility of Ketone Bodies Measurement in Inborn Errors of Metabolism. *Annals of Pediatric Endocrinology & Metabolism*.

[15] Mitra A., De Souza C., Bhatt S., Maheshwari R., Agarwal R. (2018). Approach to Hypoglycemia in Childhood. *Pediatrics in Review*.

[16] Santer R., du Moulin M., Shahinyan T. (2021). Fructose-1,6-Bisphosphatase Deficiency: A Rare Disorder of Gluconeogenesis–Clinical, Biochemical and Genetic Features in 28 Patients. *Orphanet Journal of Rare Diseases*.

[17] Ni Q., Tang M., Chen X. (2024). Fructose-1,6-Bisphosphatase Deficiency: Estimation of Prevalence in the Chinese Population and Analysis of Genotype-Phenotype Association. *Frontiers in Genetics*.

[18] Dalili S., Pirsaraei N. S., Sharifi A. (2023). Intrafamilial Phenotypic Variability due to a Missense Pathogenic Variant in the FBP1 Gene. *Iranian Journal of Medical Sciences*.

[19] Pinto A., Alfadhel M., Akroyd R. (2018). International Practices in the Dietary Management of Fructose 1-6 Biphosphatase Deficiency. *Orphanet Journal of Rare Diseases*.

